# 微流控芯片系统在循环肿瘤细胞分离检测中的应用进展

**DOI:** 10.3724/SP.J.1123.2021.07009

**Published:** 2022-03-08

**Authors:** Rongkai CAO, Min ZHANG, Hao YU, Jianhua QIN

**Affiliations:** 1.中国科学院大连化学物理研究所, 辽宁 大连 116023; 1. Dalian Institute of Chemical Physics, Chinese Academy of Sciences, Dalian 116023, China; 2.中国科学院大学, 北京 100049; 2. University of Chinese Academy of Sciences, Beijing 100049, China

**Keywords:** 微流控芯片, 循环肿瘤细胞, 分离检测, microfluidics, circulating tumor cells (CTCs), separation and detection

## Abstract

循环肿瘤细胞(CTCs)的分离分析一直是肿瘤相关研究中的热点方向,作为液体活检的重要标志物之一,其在外周血中的含量与癌症病发状况密切相关。然而人体血液中CTCs的含量非常低,通常来说仅有0~10个/mL,因此在开展临床血液样本中CTCs的检测前,往往需要对样本进行前处理,以实现CTCs的分离和富集。微流控芯片技术凭借样品消耗少,分离效率高,易于自动化和集成化等特点,在CTCs分离分析研究中具有诸多优势。近年来,利用微流控芯片开展CTCs分离检测的研究进展迅速,多种技术原理和检测方法相继出现。从技术原理角度进行区分,可分为生物亲和法和物理筛选法。生物亲和法主要依赖抗原抗体相互作用,或核酸适配体与靶标的特异性结合,该方法选择性高,但效率和捕获率偏低。物理筛选法则主要依据细胞本身的物理性质,诸如尺寸、密度和介电性质等差异实现分离。例如,可通过芯片微结构对CTCs进行阻隔或捕获,通过外加物理场(声、电、磁)辅助分选,也可以利用微观尺度流体力学作用对混合细胞进行筛分。物理筛选法一般通量较高,但往往分离纯度较低。同时,利用微流控芯片的集成优势将两种方法相结合,往往能得到更好的分离效果。除了以CTCs作为直接目标的正向富集外,还可以采取反向富集的策略,通过将作为干扰项的白细胞等作为靶标进行选择性地去除,可以避免直接筛选方式对CTCs细胞活性产生的影响。该文概述性介绍了利用微流控芯片开展循环肿瘤细胞分离检测的技术原理、芯片原位检测方法和研究进展,并结合现阶段存在的问题对其未来发展趋势予以展望。

## Abstract (en)

The isolation and analysis of circulating tumor cells (CTCs) is an important issue in tumor research. CTCs in peripheral blood, which are important biomarkers of liquid biopsy, are closely related to the occurrence of cancer and are used to monitor the effect of treatment on cancer patients. However, the number of CTCs in the blood samples of cancer patients is very low, usually being present at only 0-10 CTCs/mL. Therefore, prior to the detection of CTCs, it is important to preprocess clinical blood samples for efficient separation and enrichment. With the advantages of low sample consumption, high separation efficiency, ease of automation and integration, microfluidic chips can be a suitable platform for the isolation of CTCs. In the last few years, CTC separation and detection using microfluidic chips have developed rapidly, and a variety of detection methods have been developed. According to the technical principle used, microfluidics for CTC separation can be divided into biological property-based methods and physical property-based methods. The biological property-based methods mainly depend on the interaction between the antigen and antibody, or the specific binding of the aptamer and target. These methods have high selectivity but low efficiency and recovery rates. Physical separation is based on the physical properties of CTCs such as their size, density, and dielectric properties. For example, CTCs can be blocked or captured by the microstructure in the channels of microfluidic chips, sorted by external physical fields (acoustic, electrical, magnetic), or screened by micro-scale hydrodynamics. Physical property-based methods generally have a higher flux but lower separation purity. However, the advantages of biological property-based methods and physical property-based methods can be integrated to provide microfluidic chips having better separation performance. In addition to the direct positive enrichment of CTCs, a negative enrichment strategy can also be adopted. The influence of direct screening on the activity of CTCs can be avoided by selectively removing white blood cells. In this paper, recent advances in microfluidics utilized in the isolation of CTCs, including physical and immune methods and positive and negative enrichment, are reviewed. We summarized the technical principles, detection methods, and research progress in CTC separation and detection using microfluidic chips. Developing trends in microfluidics for CTC separation and analysis are also discussed.

作为液体活检的重要标志物之一,循环肿瘤细胞(CTCs)在外周血中的含量可以用来辅助判断患者的癌症病发状况^[1,2,3]^。除此以外,CTCs对于肿瘤细胞转移行为等基础研究也具有非常重要的意义^[4,5]^。然而人体血液中的CTCs含量极其稀少,通常仅有0~10个/mL,与之相对,红细胞、白细胞和血小板的含量则分别达到5×10^9^ 个/mL、4×10^6^ 个/mL和3×10^8^ 个/mL,而且肿瘤细胞在转移过程中可以通过上皮-间质转化(EMT)和间质-上皮转化(MET)来不断地改变自身的特征^[6]^。正是由于其稀缺性和异质性,以及血液中复杂基质的干扰,CTCs的精准检测成为巨大的难题。

由于常规的光学分析手段在检出限和灵敏度上均难以达到直接检测的要求,因此通常在进行外周血中CTCs的检测之前,要通过一些样品前处理方法来实现其分离和富集。常采用的样品前处理方法可以分为物理法和化学法^[7]^,物理法主要根据细胞在物理特征上的差异来进行分离,例如膜过滤分离^[8,9,10]^和密度梯度离心^[11,12]^,就是分别依据细胞的大小和密度来完成筛选。化学法则主要依靠生物大分子的特异性识别作用^[13,14,15]^,例如抗原抗体相互作用,核酸适配体与靶标的选择性结合。

上述样品前处理方法虽然能够在不同程度上实现CTCs的分离富集,但也存在着一定的缺陷。由于这些方法都是非连续性的,在吸附、洗脱和转移的过程中难免会造成细胞的丢失,加之CTCs本身的稀缺性,很容易导致假阴性结果的产生。利用微流控芯片功能集成的特点则可以很好地解决这一问题,CTCs的捕获、释放、计数及检测等操作均可在芯片上完成,连续的自动化处理可以有效减少人为误差的干扰^[16]^。此外,微流控芯片所需要的进样量非常小,可以大大减少珍贵样品和试剂的消耗,降低检测成本。并且在微尺度下表面力的作用会明显放大,可以有效提高物质混合和反应的效率,实现快速高效的分离分析。因此,近年来多项研究尝试利用微流控芯片平台开展CTCs分离检测工作,取得了良好的效果^[17]^。本文对微流控芯片技术用于CTCs分离检测的相关研究进展进行了综述,将采用的分离方法主要分为物理筛选和生物亲和两大类,同时囊括正向富集和反向富集两种策略。此外,对于近期发展的芯片原位检测CTCs新方法也进行了介绍。

## 1 CTCs分离芯片研究进展

作为商品化较为成功的CTCs分离检测系统,强生公司的CellSearch产品采用的是基于上皮细胞黏附分子(EpCAM)抗体特异性识别肿瘤细胞的方法^[18]^,类似的方法在CTCs分离芯片中也被广泛使用,可以视作利用生物亲和作用进行CTCs分离富集的代表。另一方面,依据细胞在物理性质方面的差异,无须生物标志物的条件下即可实现CTCs的筛选,其中有无外力介入的被动分离方法,例如利用微尺度下流体力学中的惯性效应^[19,20,21]^和黏弹性效应^[22,23]^来进行筛分。也有外加物理场的主动分离方法,诸如介电泳^[24,25,26]^、表面声波^[27,28]^和光镊技术^[29]^等。除了直接对CTCs进行特异性识别实现正向富集外,也可以通过选择性结合诸如白细胞等干扰,再将其排除,从而达到反向富集的效果^[30,31,32]^。

### 1.1 基于生物亲和作用的正向富集

由于利用EpCAM抗体来特异性识别肿瘤细胞的方法取得了不错的成效,许多研究者就采用该方法构建微流控芯片来筛选CTCs^[33,34,35]^。通过将EpCAM抗体修饰在芯片通道表面上,即可利用抗原抗体的结合作用来捕获CTCs。但由于微通道中的流体通常呈低雷诺数的层流流动,物质的混合仅能依靠横向扩散,且只有接触通道表面上的CTCs才可能被捕获,扩散速率低和接触面积小的问题导致CTCs的净捕获率较低。

为了解决这些问题,一般采用在芯片上增加微结构或对通道形状进行特殊设计的方法。Stott等^[36]^设计了一种人字形脊芯片,即在芯片通道上表面增添了长短臂交错的鱼骨状槽道。根据流体力学模拟,这种结构能够使流体通过沟槽附近时产生侧向的二次流,由此引起的涡流可以明显增大混合效率,促使细胞与通道及沟槽表面充分接触,从而提高对CTCs的捕获率。采用人字形脊芯片进行全血样分析,在1.2 mL/h的流量下得到的CTCs加标回收率为91.8%±5.2% (*n*=6),与直通道样式的芯片相比有了显著的提高。Sun等^[37]^采用微柱阵列结构的芯片,构建流动域和捕获域的功能化分区,并在捕获域微柱上修饰EpCAM抗体,即便在整体流速较高的情况下,依然能保证CTCs的高捕获率,大大提高了芯片在实际应用时的通量。

然而芯片结构的改善涉及加工工艺的问题,对于微结构和微槽道的精密刻蚀,需要昂贵设备与专业技能的支持。为了避开这一难题,同时又能保证CTCs的捕获率,以聚合物分子链或天然生物膜取代微结构的方法被提出。Yu等^[38]^采用静电纺丝技术在玻璃芯片基底上制备了呈随机排列形貌的聚乳酸-羟基乙酸共聚物(PLGA)纳米纤维(见图1a), PLGA具有良好的生物相容性,利用其带有末端羟基的性质,可以很方便地通过缩合反应连接上生物素,再借助生物素与亲和素的相互作用,即可修饰上EpCAM抗体。随机排列的PLGA纳米纤维构成了复杂的网状结构,不仅可以有效防止因直接碰触通道表面而导致的非特异性吸附,也大大提高了反应接触面积,从而增加了CTCs的净捕获率。通过加标回收的方式,测得该芯片对人体全血样本中CTCs的捕获率可达到90%。Jan等^[39]^利用相似原理,采用修饰有抗体的杂化硅纳米线结构来捕获CTCs,并先后结合激光捕获显微分离技术、温度控制及免疫竞争等方法研发了4代CTCs分离芯片,实现了CTCs捕获后的单细胞分析和可控释放。Sun等^[40]^则以带有目标肿瘤细胞印迹的聚二甲基硅氧烷(PDMS)膜为模板,通过在印迹区域修饰EpCAM抗体来捕获目标CTCs(见图1b),细胞印迹的引入可以提高捕获的特异性,减少假阳性结果的产生,改变印迹数目还可以控制芯片的捕获容量。Wu等^[41]^采用EpCAM适配体功能化的白细胞纳米囊泡来修饰芯片,构建了多价纳米流体界面(见图1c),相较于普通的适配体功能化芯片,不仅捕获效率提高了7倍,而且由于天然生物膜可减少对血细胞的非特异性吸附,捕获纯度也显著提升。Cheng等^[42]^将带有金纳米颗粒涂层的3D导电支架集成在微流控芯片中,Au-S作用便于抗体功能化修饰和电化学释放,支架的大孔结构可以促进流体混合,高密度的纳米金颗粒界面则能够提高与细胞的相互作用。该系统不仅能够可逆捕获/释放CTCs,还可以通过非特异性吸附损耗白细胞,提高回收的CTCs纯度。

**图1:**
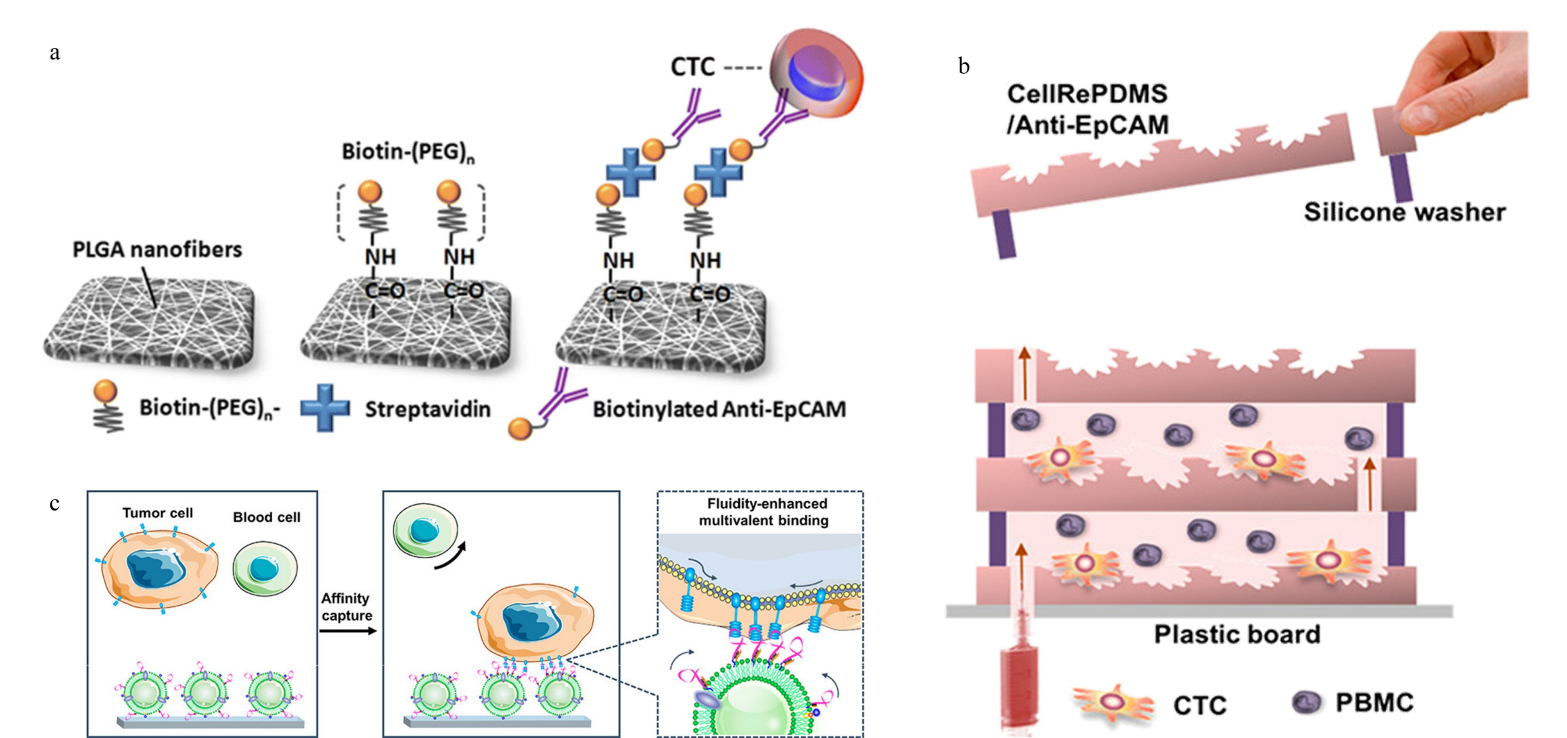
基于生物亲和原理的循环肿瘤细胞分离芯片

然而单纯依赖EpCAM抗体的特异性识别作用来进行CTCs捕获具有一个较大的缺陷,由于高度转移性的CTCs可能经历上皮-间质转化,引发EpCAM表达明显下调,最终导致这部分CTCs被忽略,从而产生假阴性结果。Liao等^[43]^为了精确筛选出这部分EpCAM表达下调的CTCs,结合免疫捕获芯片和光诱导介电泳(ODEP)技术进行了实验探究。首先采用EpCAM抗体来标记肿瘤细胞、CD45抗体标记白细胞以及钙黄绿素乙酰氧基甲酯(Calcein AM)标记所有活细胞,则正常的CTCs表达为Calcein AM^pos^/EpCAM^pos^,干扰最大的白细胞表达为Calcein AM^pos^/CD45^pos^,经历了EMT的CTCs表达为CD45^neg^/EpCAM^neg^。然后利用ODEP技术,根据荧光显色情况的差异,即可在芯片主干通路与分支通路的交叉口实现CD45^neg^/EpCAM^neg^细胞的分选,且分离纯度高达100%。该方法不仅可以用于捕获因经历过EMT而被遗漏的CTCs,同时也为研究低EpCAM表达的肿瘤细胞提供了有效的手段。Wu等^[44]^在PLGA纤维化基底上同时修饰了EpCAM和N-钙黏蛋白两种适配体,在确保捕获EpCAM表达正常的CTCs同时,也能够减少EpCAM低表达CTCs的遗漏,可以提高整体捕获率并减少假阴性结果的产生。

### 1.2 基于物理筛选方法的正向富集

物理筛选法依据的主要是细胞本身物理性质的差异,相较于生物亲和法而言,实验操作往往更加简单,无须进行化学修饰和生物标记,因此对细胞活性影响较小。效仿宏观体系下的物理分离方法,微流控芯片上的物理筛选法通常依赖于外加物理场的诱导,或是直接依据孔筛原理利用细胞大小差异来分离。Jahangiri等^[45]^依据不同种细胞间电极化常数的差异,通过在芯片上施加低频交流电场,完成了对不同种乳腺癌CTCs和血细胞的分离。Gascoyne等^[46]^则在外加交流电场的条件下,利用介电场流分离(depFFF)的方法对血液中的CTCs进行分离筛选,依据细胞介电特性等的差异实现了CTCs的富集,细胞捕获率超过90%,且对10 mL的临床样本进行处理仅需15 min,分离效率远高于生物亲和法。Wu等^[47]^利用表面声波分离原理,通过外加声场和表面声波传感器,依据大小、密度和形状不同的细胞在驻波场中的排列分离,实现了外周血中CTCs的分选(见图2a),在7.5 mL/h的通量下可得到86%以上的回收率。

**图2:**
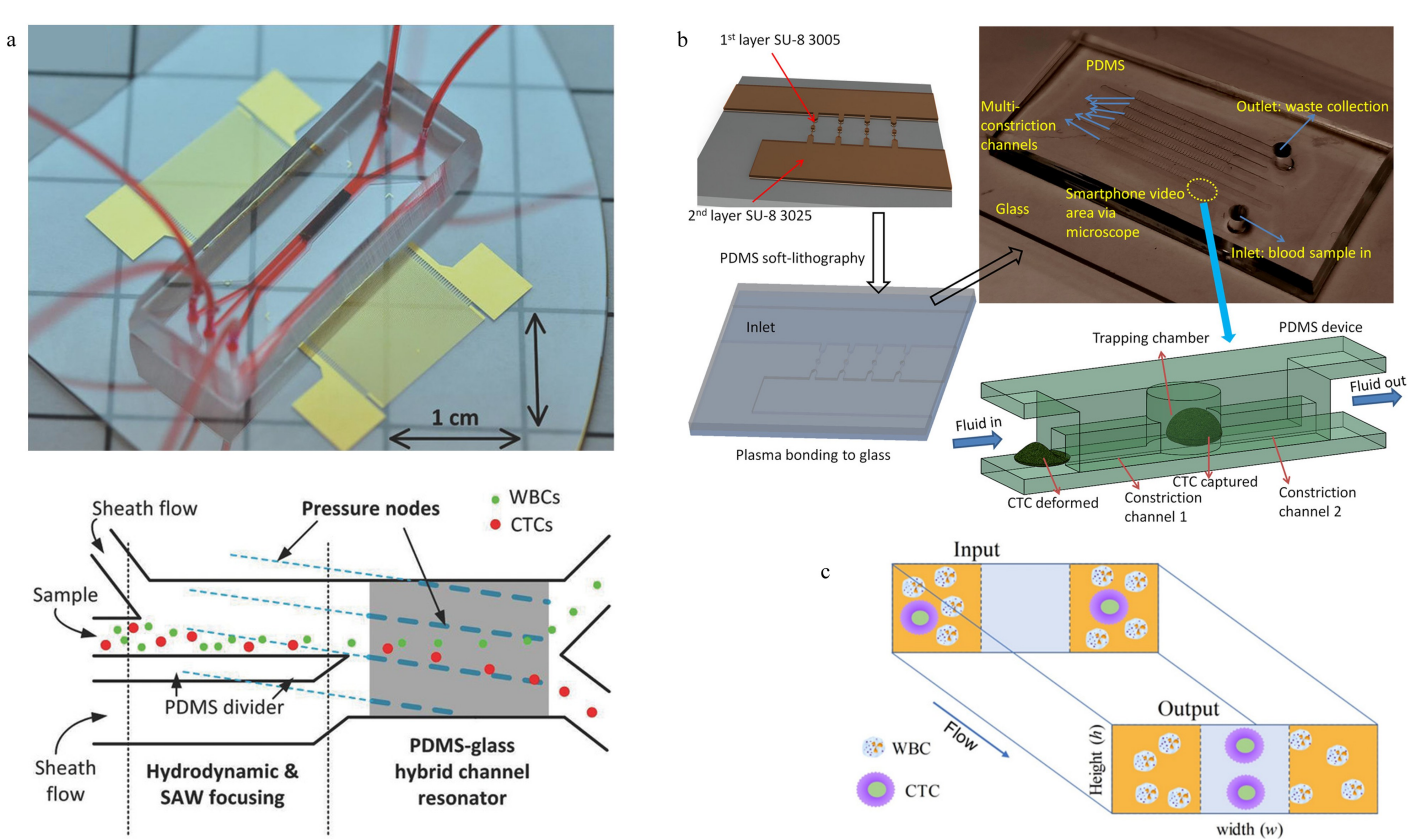
基于物理筛选方法的循环肿瘤细胞分离芯片

然而外加场源在芯片上的集成化是比较困难的,因此Lin等^[48]^根据孔筛过滤原理设计了一种非常简单的CTCs分离芯片。通过调控滤孔的大小,依据细胞尺寸的差异即可完成CTCs的分离,实验所得回收率超过90%。Chen等^[49]^基于仿生脾窦微结构构建了孔筛式芯片系统,裂隙结构的滤孔相对于传统的圆形结构具有更低的流动阻力,通过流速和狭缝宽度的优化,可以在实现高效分离的同时,保证CTCs的高细胞活性。但是由于白细胞与CTCs在大小上基本相当,该方法筛选精度较低,可能产生假阳性结果。此外,在细胞流动通路上设置滤孔容易因堵塞导致负压增大,从而影响分离效率。为了解决上述问题,同时保留方法的便利性,研究人员对依据细胞尺寸实现CTCs筛选的方法进行了优化,将起过滤作用的微柱结构或捕获腔室设置在侧向,来避免由于细胞堵塞所引起的主干通路负压过大的问题。Ren等^[50]^依据细胞尺寸和变形性的差异设计了一种高通量的CTCs捕获芯片(见图2b)。该芯片具有多条通道,且通道间由多排微型收缩管相连通,微型收缩管中有依据CTCs尺寸设计的捕获腔。血液在流经主通道与微型收缩管交汇处时,由于表面张力所产生的毛细作用会驱使流体通过收缩管,此时CTCs会被困在捕获腔中,血液中其他组分则能顺利通过,继而进入相邻通道。为了保证较高的CTCs捕获率,该过程可在多条通道间重复进行。采用该芯片对每毫升含有50个前列腺癌细胞的小鼠全血样品进行处理,当通道数达到6条时,CTCs捕获率可超过95%。Liu等^[51]^将过滤的概念与确定性侧向位移原理(DLD)相结合,设计了级联DLD微柱阵列芯片,能够在1 mL/min的高通量下实现96%的CTCs回收率,同时能够剔除99.99%的白细胞。

依据细胞尺寸进行CTCs筛选的芯片中通常会有许多起物理阻隔作用的微结构,这些精密结构的加工往往是比较困难的。随着微尺度下流体力学理论的发展,基于微流体中颗粒运动规律的研究,出现了无须任何微结构,仅仅通过对流体的调控便可实现CTCs分离富集的方法。Kulasinghe等^[52]^依据微尺度下的惯性效应设计了一种结构十分简单的方形通道芯片(见图2c)。在方形管道中由于Dean涡与惯性升力的共同作用,在管道长边中点附近会产生平衡位点,直径大的细胞会优先在该位点中心聚集。因此CTCs会富集在靠近通道中心处,其他组分则排布在外侧。该芯片中没有微结构或捕获腔来增加负压,因此在处理通量上能有更高的突破,此外,由于无任何其他外部作用的影响,细胞能够更好地保留其生理活性和形态特征。Lim等^[53]^同样依据惯性效应,通过在T形通道处引入切向流,筛去靠近管壁侧的血细胞,从而实现CTCs的分离。Zhang等^[54]^与Tian等^[55]^则利用非牛顿流体中的黏弹性效应完成了对CTCs的分选,当采用低黏度、无剪切稀化的流体时可达到与惯性效应相近的效果。而且该方法的理论汇聚模式较为简单,便于进行更为准确的数值模拟分析。Zhu等^[56]^以聚合物薄膜为材料,通过拼图技术构建了梯形通道的螺旋状微流控芯片,利用梯形通道中的惯性力和Dean涡流综合作用,可以在3 mL/min的高通量下实现CTCs的分选,实验回收率为90%~94%。Lu等^[57]^设计的微流控芯片系统将微尺度流体力学和孔筛原理相结合,先通过惯性效应完成对血细胞的初步分离,再利用三角微柱阵列实现对CTCs的捕获,该方法在保证94.8%的高捕获率的基础上,通量可以达到40 mL/h。

上述CTCs分离芯片大都只关注于单个细胞,并没有针对CTCs簇设计专门的选择性分离方法。考虑到CTCs簇的研究对于肿瘤学也具有非常重要的意义,Sarioglu等^[58]^设计了含有三角形微柱结构的芯片对CTCs簇进行高精度分选。当CTCs簇流经三角微柱时,由于胞间连接作用会在其顶点处被捕获,单个细胞则会直接沿着微柱侧腰面通过,该方法要求流速不能过高,否则较大的剪切力会破坏CTCs簇的结构致使捕获失败。采用该芯片对实际样品进行分离,确定了CTCs簇的异质性,并发现其中可能含有肿瘤相关巨噬细胞(TAMs),该发现对TAMs与CTCs间相互作用的研究具有重要意义。

### 1.3 生物亲和与物理筛选相结合的正向富集

总体而言,基于生物亲和作用和物理筛选方法的CTCs分离芯片各有其优势。前者选择特异性更强,后者分离效率更高。同时二者也都存在不足之处,例如生物亲和法依赖于外源性标记,往往会影响CTCs细胞活性,物理筛选法分离精度较低,容易产生假阳性结果。因此研究人员尝试将两种方法相结合^[59,60,61]^,取长补短以实现更好的分离效果。Chen等^[62]^采用侧向微柱阵列结构的芯片,在微柱上修饰了EpCAM抗体,结合孔筛原理与免疫亲和作用来提高CTCs的捕获效率,同时由于主流场方向无阻塞,可以保证较高的通量(见图3a)。Su等^[63]^利用相同的原理,在此基础上引入表面功能化修饰的氧化锌微球,大大提高了有效捕获面积(见图3b)。相似地,Chen等^[64]^先利用修饰了EpCAM抗体的磁珠去特异性结合CTCs,再让样品流经椭圆微柱阵列区,吸附了磁珠的CTCs变形性减弱,且由于外加磁场的作用不能通过窄间隙阵列,从而达到分离效果。利用该方法对11例不同癌症临床样本进行分析,捕获率超过90%,且即便在高流速下,细胞存活率也能达到96%。Song等^[65]^则结合确定性侧向位移原理设计了多价核酸适配体纳米微球(AuNP-SYL3C)修饰的芯片(见图3c),尺寸和弹性不同的细胞在流经转角三角形微柱阵列区时,会因与微柱的碰撞选择不同的路径,通过调控微柱大小和间距,可使CTCs碰撞微柱时产生侧向位移,其他血细胞则沿原路径流出。此外,微柱上修饰有AuNP-SYL3C,通过核酸适配体多价效应可以大大增强结合力,从而显著提高对CTCs的捕获性能,与单价核酸适配体修饰芯片相比,该方法的捕获效率提高了3倍以上。

**图3:**
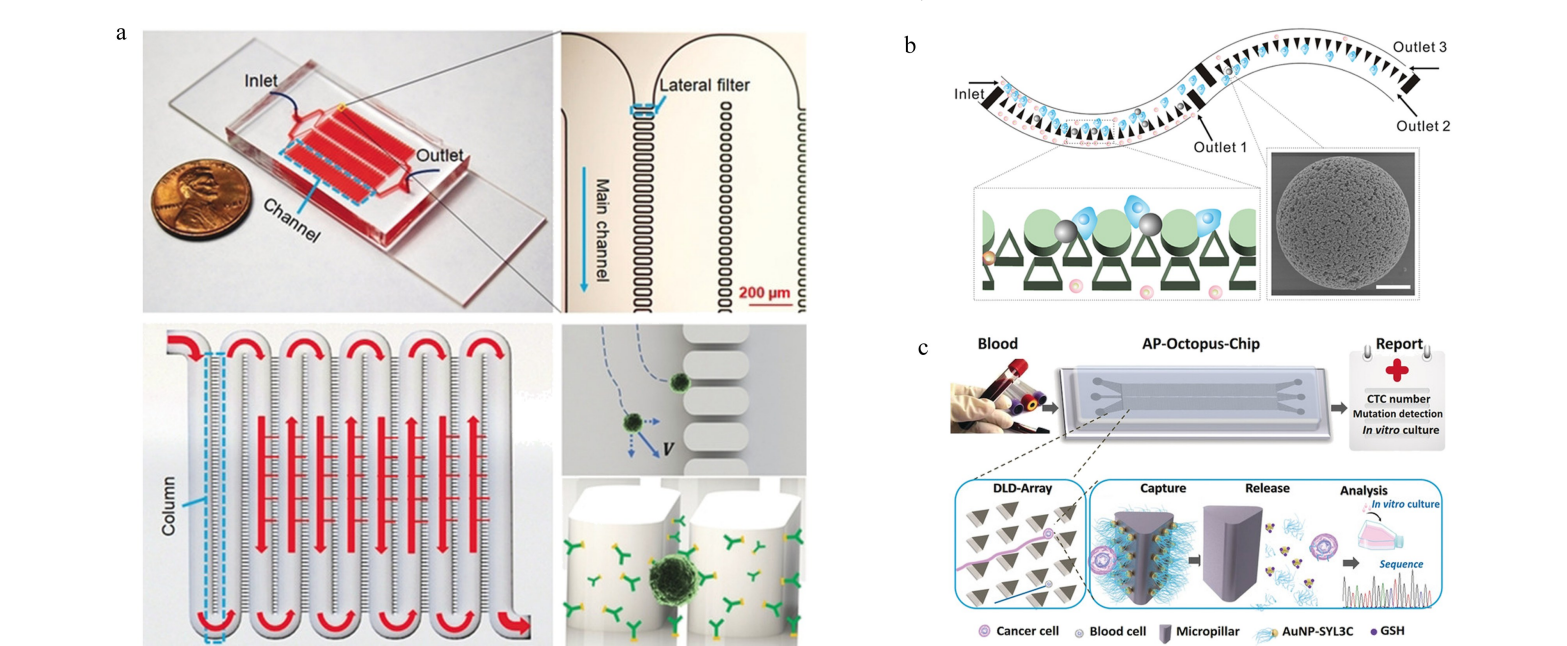
基于综合作用的循环肿瘤细胞分离芯片

### 1.4 反向富集

由于白细胞与CTCs在尺寸上差别不大,在对血液中的CTCs进行分选时,白细胞往往是干扰最大的因素。因此可以通过选择性分离白细胞,以达到CTCs富集的目的,也就是反向富集。采用反向富集的策略不仅能有效实现CTCs的分离,而且对于因EMT导致EpCAM表达下调的CTCs,甚至于非上皮性肿瘤细胞都可进行富集,同时也能够避免直接标记对CTCs细胞活性产生影响。Chu等^[66]^利用3D打印构建了功能化分区的微流控系统(见图 4a),

**图4:**
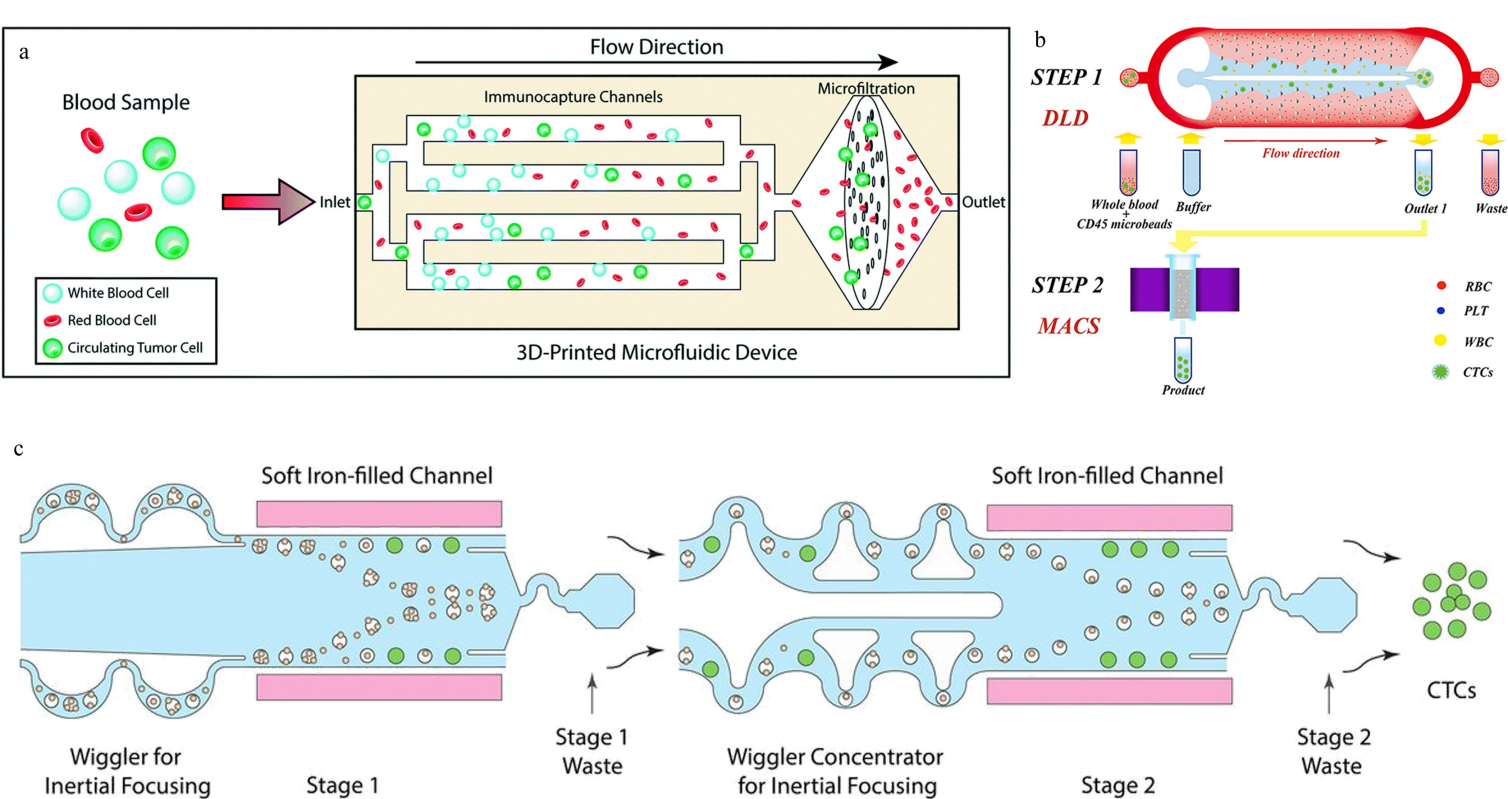
基于反向富集策略的循环肿瘤细胞分离芯片

血液样本先流经修饰有CD45抗体的免疫捕获区,选择性筛去白细胞,再通过3 μm孔径的滤膜,除去小体积的红细胞和血小板,从而实现CTCs的分离。Karabacak等^[67]^设计了集成双芯片的分离体系,先通过DLD和惯性效应两种物理筛选法将白细胞和CTCs从血液中快速分离出来,再用修饰了CD45和CD66b复合抗体的磁珠来选择性结合白细胞,继而在外加磁场的诱导下实现白细胞与CTCs的精确筛分。该方法在保证高CTCs捕获率的情况下,还能达到较高通量,处理8 mL血液样本仅需2 h。Wang等^[68]^采用了相似的DLD-MACS(免疫磁珠分选)方法(见图 4b)对肝癌患者临床样本进行分析,在60 μL/min的流量下得到CTCs的捕获率为85.1%±3.2%,且实验表明该方法对于EpCAM低表达的肿瘤细胞依然有很好的分离效果。Mishra等^[69]^设计的CTC分离芯片将免疫磁分选与惯性效应相结合(见图 4c),利用修饰多种抗体的免疫磁珠来标记白细胞,在外加强磁场和流体调控下可以实现CTCs和白细胞的高效分离,该芯片对CTCs的富集作用可以达到10^5^倍,通量高达168 mL/h。基于微流控技术的各种CTCs分离方法汇总详见表1。

**表1:** 基于微流控技术的循环肿瘤细胞分离方法

Methods	Sample	Throughput/ (mL/h)	Recovery/ %	Depletion of WBCs/%	Viability/ %	Ref.
Biological property-based methods						
Antigen modified microstructure	PC3 cells spiked into whole blood	1.2	~92	14 (purity)	~95	[36]
Aptamer functionalized nanointerface	Cancer cells suspended in buffer	0.35	~91	>99.9	~98	[41]
Antigen coated 3D scaffold	MCF-7 cells and WBCs suspended	6	~93	N/A	~91	[42]
	in PBS					
Physical property-based methods						
Dielectrophoretic field-flow	MDA-MB-435 and PBMN cells	90	~92	>95	~90	[46]
fractionation	suspended in buffer					
Surface acoustic wave	Cancer cells and WBCs suspended	7.5	>86	>97	N/A	[47]
	in PBS					
Biomimetic filtration membranes	MDA-MB-231 cells spiked into diluted	30	~90	>45 (purity)	~91	[49]
	blood					
Deterministic lateral displacement	A549 and K562 cells spiked into	60	>96	>99.99	>98	[51]
	diluted blood					
Inertial focusing	A549 cells spiked into lysed blood	180	~94	~79 (purity)	N/A	[56]
Integrated methods						
Lateral filter arrays with immunoaffinity	L3.6pl cells spiked into diluted blood	3.6	~95	>99.5	~84	[62]
Immunobeads integrated filter chip	MCF-7 cells spiked into lysed whole	3	>85	~40 (purity)	N/A	[63]
with inertial flow	blood					
Multivalent aptamer modified DLD-array	Cancer cells spiked into whole blood	1	~84	~99.99	~96	[65]
Negative enrichment strategy						
Immunocapture channels with	Cancer cells spiked into whole blood	0.5	>90	~96	>90	[66]
microfiltration						
DLD arrays integrated with MACS	Cancer cells spiked into whole blood	8	~97	>99.9	N/A	[67]
and inertial focusing						
MACS combined with inertial focusing	Cancer cells spiked into whole blood	168	~86	~99.97	N/A	[69]

N/A: not applicable.

## 2 芯片原位CTCs检测

对于CTCs的检测,通常采取先进行细胞染色,再用荧光显微镜观察的方法^[70]^,但该方法在灵敏度上有待提高,且重现性较差,需要手动操作和人工计数。近年来研究人员对芯片上的CTCs成像分析方法进行了优化改良,Pahattuge等^[71]^研发的SMART-Chip将CTCs分选、细胞计数和免疫荧光成像模块集成于一体,实现了对血液中CTCs分离检测的全自动化操作,避免了人为干扰。Lee等^[72]^则通过多通道荧光成像,同时表征CTCs上的雌激素受体(ER)、孕激素受体(PR)和人类表皮生长因子受体2(HER2)表达状况,完成对乳腺癌的快速诊断和分型。Wang等^[73]^在集成化的CTCs分离、免疫荧光染色和成像系统中引入了气体驱动装置,大大缩减了时间和试剂的消耗,能够在90 min内实现CTCs的捕获和识别。Shi等^[74]^在集成化的微流控系统中实现了CTCs的单细胞分离和免疫染色鉴定,以及细胞的裂解和单细胞内容物的靶向收集,不仅在单细胞水平上完成了CTCs的检测,还为后续的单细胞RNA测序奠定了坚实的基础。Wang等^[75]^反其道而行之,采用修饰CD45抗体的免疫微球来标记白细胞,通过楔形芯片完成血细胞初步分选后,可在明场显微成像下直接区分出CTCs并通过图像处理软件实现自动计数。

此外,以荧光光谱为代表,一些常见的光谱检测手段也被广泛应用在芯片上CTCs的检测中。Wu等^[76]^用同时负载有抗体和荧光编码的磁性纳米颗粒来标记CTCs上的靶标蛋白,在外加磁场下利用芯片捕获CTCs,并能通过荧光强度完成对单个CTC上表皮生长因子受体(EGFR)、HER2和EpCAM含量的表征(见图5a)。Dhar等^[77]^通过液滴微流控技术,将惯性涡流分离后得到的CTCs包裹在含有基质金属蛋白酶(MMP)反应体系的液滴中,由于目标CTCs具有很高的MMP反应活性,可由该酶促反应体系产生的共振荧光转移现象来实现对CTCs的检测和计数。Shen等^[78]^设计了一种以金膜为基底的芯片,通过免疫磁性分离的方法对CTCs进行分选,之后采用基于表面等离子共振的近红外荧光法实现对CTCs的观察和检测,由于近红外区生物样品基体光吸收和自发荧光强度很小,且表面等离子体共振效应大大增强了荧光信号强度,该方法的检测灵敏度与普通的荧光分析方法相比提高了近10倍。Cho等^[79]^采用修饰有抗体和拉曼信号分子的金纳米颗粒来标记CTCs,通过表面增强拉曼技术即可对芯片上被捕获的CTCs进行原位表征和检测。该方法不仅具有高灵敏度,还可以依据拉曼信号峰的差异区分普通的CTCs以及循环肿瘤干细胞(CCSCs)。Reza等^[80]^结合表面增强拉曼和微流控技术,在单细胞水平上实现了对CTCs多种蛋白标志物的原位动态监测,CTCs在蛋白表达水平上的异质性能够反映更多的关键信息,为癌症临床诊断和治疗提供判断依据。

**图5:**
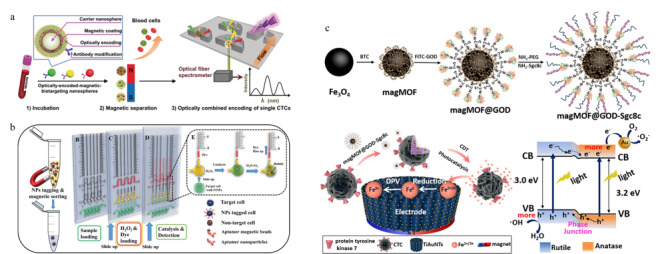
芯片原位检测循环肿瘤细胞方法

除了光学分析方法外,研究人员通过使用传感元件实现了CTCs芯片检测结果的数字化直读或可视化分析。Chen等^[81]^以具有高电子迁移率的AlGaN/GaN为材料制作场效应晶体管(FETs),在芯片上设置FETs传感器阵列,并在其表面修饰上特异性识别EpCAM的核酸适配体,从而实现了CTCs的连续捕获和计数。FETs的高跨导增益使得该生物电子传感器具有很高的检测灵敏度,实验表明该方法能够在较宽的动态范围内实现CTCs的快速自动化检测,并能够提供准确的细胞计数数据。Gao等^[82]^将采用复合免疫磁珠进行筛选的CTCs芯片与液滴数字PCR芯片相结合,通过PCR扩增来提高检测的灵敏度。Abate等^[83]^在待测血样中加入修饰有核酸适配体的铂纳米粒子(PtNPs),通过免疫磁性分离得到结合有PtNPs的目标CTCs,将其引入预载有H_2_O_2_和染料的检测芯片中,由于Pt可以催化H_2_O_2_的分解,产生的氧气会导致染料液柱的上升,根据液柱高度可以判断血液中CTCs的含量(见图5b)。Jian等^[84]^用修饰有葡萄糖氧化酶的磁性MOF纳米颗粒来标记CTCs,芯片上的TiO_2_纳米管阵列借由磁力完成对CTCs的捕获后,通过TiO_2_的光催化作用将MOF中的Fe^2+^/Fe^3+^还原为Fe^0^,利用微分脉冲伏安法得到电化学信号,即可实现CTCs的定量检测(见图5c)。

## 3 总结与展望

本文对CTCs分离微流控芯片的技术原理、分离策略和研究进展进行了综述。其技术原理主要分为物理筛选和生物亲和两大类,分离策略分为正向富集和反向富集两个方向。同时,介绍了CTCs芯片原位检测的主要技术方法和优化策略。随着微流控芯片技术的快速发展,其微尺度流体操控、微结构加工和集成传感检测能力得到极大提升,进一步推动了CTCs分离微流控芯片技术的发展。多项研究显示,以微流控芯片为平台来分离检测外周血中的CTCs,可以充分发挥芯片本身微量、高效、易于自动化和集成化的优势,最终实现对临床血液中CTCs的快速精准分析,在肿瘤早期诊断、复发与转移监测以及抗肿瘤药物评价等多个领域具有重要的应用空间。

现阶段,CTCs芯片在筛选精度和筛选效率方面仍存在较大的提升空间。针对这一挑战,由于精准与高效二者难以兼得,未来的芯片设计应该更专注于单个目标的实现。一方面,针对基础研究,应当注重于提高CTCs筛选的细胞纯度及细胞活性。可以先利用惯性效应对血液进行粗分离,筛分出尺寸较大的白细胞和CTCs。再采用液滴分选的方法,通过免疫磁性分离实现CTCs的精确筛选。液滴分选技术能够达到单细胞分析的精度,利用液滴分选进行肿瘤细胞筛选也已有文献报道^[85]^。另一方面,针对临床检测领域,研究重点则在于实现临床样本的高通量分析。可以采用电分析方法,依据不同种类细胞的比膜电容和细胞质电导率差异来设置恰当的阈值,对流经检测窗口的CTCs实现快速分析^[86]^。此外,微流控芯片技术属于多学科交叉领域,CTCs芯片的发展同时也受益于微机电系统(MEMS)、材料学、流体力学和生物医学等研究领域的技术突破。随着相关领域研究技术的发展,CTCs芯片未来有望成为肿瘤基础研究和癌症早期临床诊断的重要平台。
